# HDAC10 Inhibits Cervical Cancer Progression through Downregulating the HDAC10-microRNA-223-EPB41L3 Axis

**DOI:** 10.1155/2022/8092751

**Published:** 2022-01-19

**Authors:** Yuan Yuan Gu, Guan Nan Zhou, Yao Li, Hong Yu He, Jing Xin Ding, Ke Qin Hua

**Affiliations:** ^1^Department of Gynecology, The Obstetrics and Gynecology Hospital of Fudan University, 419 Fang-Xie Road, Shanghai 200011, China; ^2^Shanghai Key Laboratory of Female Reproductive Endocrine Related Diseases, Shanghai 200011, China; ^3^Changning Maternity and Infant Health Hospital, East China Normal University, Shanghai, China; ^4^Department of Urology, Gongli Hospital of Shanghai Pudong New Area, 219 Miao-Pu Road, Shanghai 200135, China; ^5^Department of Intensive Care Unit, Zhongshan Hospital, Fudan University, 180 Feng-Lin Road, Shanghai 200011, China

## Abstract

**Background:**

Although the tumorigenesis of cervical cancer (CC) has been widely investigated and recognized, the study of the systematic impact of histone deacetylase 10 (HDAC10), microRNA, and downstream molecular mechanisms in CC is still limited. Herein, cervical cancer, precancer lesions, and normal cervical tissues were collected to test the expression level of HDAC10, miR-223, and EPB41L3. The mechanism of HDAC10, miR-223, and EPB41L3 was interpreted in cervical cancer cells after HDAC10, miR-223, or EPB41L3 expression was altered.

**Results:**

HDAC10 was poorly expressed in cervical cancer and precancer lesions, while miR-223 was highly expressed in cervical cancer. HDAC10 bound to miR-223, and miR-223 targeted EPB41L3. HDAC10 depressed the invasion property and tumorigenesis of cervical cancer via downregulating miR-223 and subsequently targeting EPB41L3.

**Conclusion:**

The study clarifies that HDAC10 inhibits cervical cancer by downregulating miR-223 and subsequently targeting EPB41L3 expression, which might provide a new insight for management upon cervical cancer and precancer lesions.

## 1. Background

Uterine cervical cancer, which is acknowledged to be initiated by a persist high-risk type of human papillomavirus (HPV) infection, is one of the most common gynecologic malignancies worldwide [1, 2]. While the multistage development of cancer biogenesis from cervical squamous intraepithelial lesion (SIL) to cancer was widely illustrated, the impact of posttranslational modifications of histone proteins on cervical cancer tumorigenic process is poorly understood. Furthermore, while the current first-line treatment strategy for cervical carcinoma includes surgery [3], chemotherapy or radiotherapy [4], and emerging immunotherapy [5] (even though the effectiveness and the application are still limited), the treatment strategy for cervical carcinoma needs further development for the reason of the plateau. Consequently, well recognition of the mechanism of cervical cancer tumorigenesis and metastasis at the molecular level contributes to figure out cervical cancer progression and further develop potential effective therapies.

Accumulating evidence indicates that abnormal regulation of acetylation processes plays a vital role in tumor carcinogenesis [6]. Histone acetylation is acknowledged as a kind of biological process. Histone acetylation is mediated by two kinds of enzymes: histone acetyl transferases (HATs) and histone deacetylases (HDACs) [7]. The histone deacetylase (HDAC) family of transcriptional corepressors, which have been central in the understanding, have emerged as important regulators of cancer biogenesis. In fact, HDACs play roles in modulating the cellular differentiation and regulating the angiogenic process through various molecular pathways (including modulating the transcription of genes and regulating the expression level of cellular proteins). Several studies reported that HDAC10 was related to the progression and biological features in cervical cancer: HDAC10 inhibits the metastasis of cervical carcinoma through downregulating the expression of MMP2 and MMP9 [8]; HDAC10 promotes the cervical cancer progression in vitro while the mechanism is still unclear and remains to be further explored [9].

MicroRNAs are regarded as functional molecules in tumor proliferation and invasion. It is previously demonstrated by several studies that miR-223 takes part in the biogenetic process of diverse carcinomas (including lung carcinoma [10] and prostate carcinoma [11] as well as esophageal squamous cell carcinoma [12]) in respect of the proliferation ability and invasion ability.

EPB41L3, one of the band 4.1 family proteins, is a kind of cytoskeletal protein [13]. EPB41L3 is well acknowledged that it could act as a kind of protein linker between the actin cytoskeleton and transmembrane proteins to maintain the cellular homeostasis. Recent studies have shown that EPB41L3 acted as a key factor in regulating cell-to-cell communication including in the cell adhesion, cell motility, and cell morphology in ESCC [14, 15], lung carcinomas [16], breast carcinomas [17], and some other tumors [18]. Intriguingly, several studies have reported that miR-223 could regulate the cellular biology via targeting EPB41L3 [19], resulting in cell migration and invasion. It was well reported that EPB41L3 plays vital role in the progression of cervical cancer [20] and oropharyngeal cancer [21]. Furthermore, the epigenetic alternations of EPB41L3 is correlated to the severity of cervical lesions [22]. Also, the EPB41L3 is selected as one of the biomarkers in the cervical cancer screening kits. Thus, EPB41L3 plays a vital role in regulating the pathophysiological process of cervical carcinoma. However, how the crosstalk network of HDAC10, miR-223, and EPB41L3 regulates the progression of cervical cancer is still unclear. The purpose of this study is to explore the effects of HDAC10/miR-223/EPB41L3 axis in cervical cancer.

## 2. Results

### 2.1. HDAC10 Is Downregulated in Cervical Carcinoma and Relevant to the Severity of the Cervical Lesions

**Figure 1:**
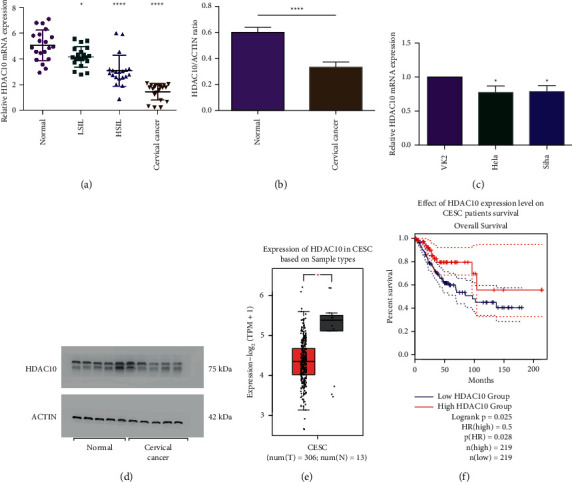
The expression level of HDAC10 is relevant to the severity of lesions and downregulated in cervical carcinoma. (a) Detecting HDAC10 mRNA expression level in cervical cancer, HSIL, LSIL, and normal tissues by RT-qPCR. (b) Quantification of the protein expression level of HDAC10 in cervical carcinoma tissues and normal tissues. (c) Expression level of HDAC10 in cervical carcinoma cell lines (HeLa and Siha) and VK2 cells. (d) Expression level of HDAC10 protein in cervical carcinoma tissue and normal tissues. (e) Expression of HDAC10 in tumor and normal tissues based on the CESC-TCGA database. (f) The overall survival analysis among women with different HDAC10 expression levels based on the CESC-TCGA database.

For clarification of HDAC10-mediated mechanism in the progression of cervical cancer, the current study detected the expression level of HDAC10 in 20 cervical cancer cases, 20 LSIL cases, 20 HSIL cases, and normal cervical tissues (Table 1). Then, we conducted the RT-qPCR assay (Table 2) and western blot assay to analyze the HDAC10 expression level in cervical cancer in both the tissue and the cells. In Figure 1(a), HDAC10 was lowly expressed in precancer lesions and carcinoma tissues when compared to normal cervical tissues, and the expression level is relevant to the severity of lesions. This result means that cervical scraping samples with more severity tended to express a lower level of HDAC10 (Figure 1(a)). The results manifested that the protein expression level of HDAC10 was low in cervical cancer tissues (Figures 1(a), 1(b), and 1(d), Figure S1) and cells (Figure 1(c)). Furthermore, we conducted the analysis about the HDAC10 expression in cervical cancer via the endocervical adenocarcinoma (CESC)-TCGA database. The expression level of HDAC10 was low in cervical cancer (Figure 1(e)). Also, we conducted the analysis about the survival prognosis in cervical cancer patients. As depicted in Figure 1(f), the downregulated expression level of HDAC10 was relevant to the lower survival rate and inferior prognosis.

### 2.2. HDAC10 Depresses the Viability and Colony-Forming Ability of Cervical Cancer

**Figure 2:**
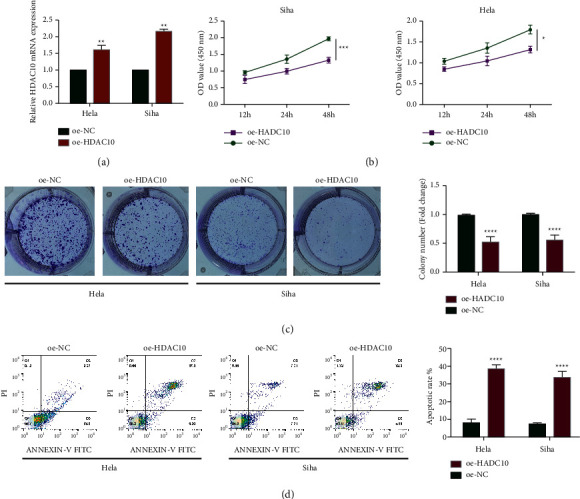
HDAC10 depresses CC cell viability and colony-forming. (a) Expression level of HDAC10 in cervical cancer cells. (b) Measurement of cervical cancer cell viability. (c) Colony-forming abilities of cervical cancer cells. (d) Apoptosis of cervical cancer cells after different treatment.

For exploring the biological functions of dysregulated HDAC10 upon cervical cancer cells, we analyzed the cell viability and colony-forming ability of cervical cancer cells after the treatment of overexpressed HDAC10 (Figure 2(a)). As shown in Figure 2(b), cervical cancer cells with overexpressed HDAC10 exhibited the impaired cell viability when compared with nonoverexpressed HDAC10 cervical cancer cells. Similarly, colony-forming ability was inhibited by the upregulation of HDAC10 (Figure 2(c)), and the apoptosis was enhanced by upregulated HDAC10 in HeLa cells and Siha cells (Figure 2(d)).

### 2.3. HDAC10 Inhibits the Invasion and Migration

**Figure 3:**
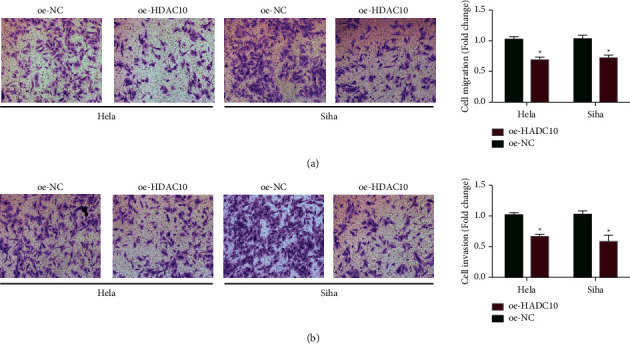
HDAC10 inhibits invasion and migration of cervical carcinoma in vitro and in vivo. (a) Measurement of cervical cancer cell migration ability. (b) Measurement of cervical cancer cell invasion ability.

**Figure 4:**
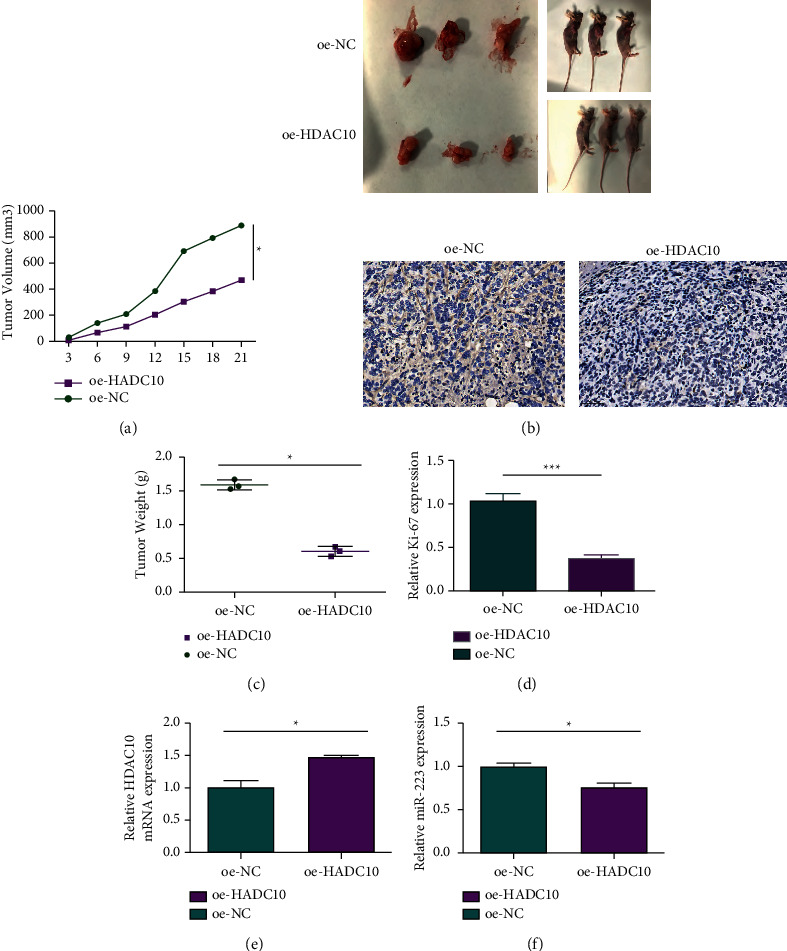
HDAC10 inhibits cervical cancer tumorigenesis in mice. (a) Xenografted tumor volume changes in two experimental groups. (b) Photomicrographs showing mice and xenograft tumors resected from the mice in the two experimental groups of the xenograft experiments. (c) Xenografted tumor weight changes in two experimental groups. (d) Representative photomicrographs showing immunohistochemically stained sections of xenograft tumor tissues stained with Ki-67. (e) Detecting HDAC10 expression level in excised tumor tissues by RT-qPCR. (f) Expression level of miR-223 in excised tumor tissues.

HeLa cells and Siha cells overexpressed with/without overexpressed HDAC10 (oe-HDAC10 and oe-NC) were tested through the transwell assay. As depicted in Figures 3(a) and 3(b), the upregulation of HDAC10 inhibited the migration ability and the invasion ability of cervical cancer cells. Furthermore, as demonstrated in Figure 4(b), the transfected HeLa cells were collected and subsequently injected into the back of mice subcutaneously; the tumor volume (Figure 4(a)) is smaller and the tumor weight is lighter in the cancer cells treated with the upregulation of HDAC10 (Figure 4(c)). The Ki-67 expression level is lower in oe-HDAC10 group when compared with the oe-NC control group, which means that the upregulation of HDAC10 inhibited the proliferation of cervical carcinoma (Figure 4(d)). According to the assessment upon the mice tumor tissues, overexpressed HDAC10 resulted in increased expression level of HDAC10 and decreased miR-223 expression levels (Figures 4(e) and 4(f)). In conclusion, HDAC10 inhibited cervical cancer.

### 2.4. HDAC10 Suppresses the miR-223 Expression via Bonding to miR-223 Promoter

**Figure 5:**
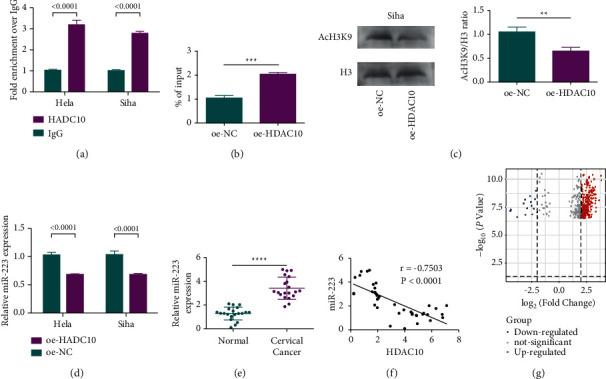
HDAC10 binds to the miR-223 promoter and suppresses the miR-223 expression. (a) Detecting HDAC10 enrichment on the miR-223 promoter in cervical cancer cells by ChIP assay. (b) Detecting miR-223 expression level in cervical cancer cells after HDAC10 overexpression by RT-qPCR. (c) Expression level of miR-223 in cervical cancer and normal tissues. (d) Analysis of the correlation between HDAC10 and miR-223 in cervical cancer tissues. (e) Analysis of relative expression of miR-223. (f) The analysis of the relationship between miR-223 and HDAC10. (g) Analysis of different miRNA expression by using the volcano plot.

It is well acknowledged that HDACs were linked to different pathways. HDACs could be recruited to the miRNAs promoter, for example, HDAC2 was reported in esophageal squamous cell carcinoma to suppress the expression level of miR-182 via bonding to the miR-182 promoter and also inhibit miR-133 in neuroblastoma [23, 24]. To further figure out the HDAC10 functioned in miR-223, the chromatin immunoprecipitation (ChIP) assay was conducted, and the results showed that HDAC10 was recruited to the miR-223 promoter specifically both in Siha and in HeLa cells (Figure 5(a)). Furthermore, it was demonstrated that the upregulation of HDAC10 in HeLa and Siha cells resulted in a lower miR-223 expression level (Figures 5(b), 5(c), and 5(d)). Moreover, the results showed that the high expression level of miR-223 was found in cervical cancer tissues (Figure 5(e)). Meanwhile, the low expression level of miR-223 and the expression level of HDAC10 are depicted in Figure 5(f). These above results were in line with the results of volcanoes mapping based on the microarray analysis of miRNA expression, which suggests that the miR-223 expressed higher in cervical cancer cells with high HDAC10 (Figure 5(g)).

### 2.5. miR-223 Targets EPB41L3 in Cervical Cancer

**Figure 6:**
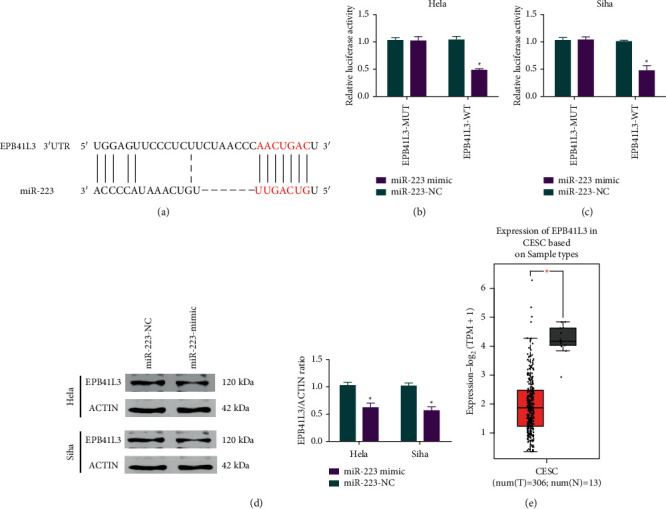
miR-223 targets EPB41L3 in cervical cancer. (a) Analysis of the binding site of miR-223 and EPB41L3 by using TargetScan software. (b) Analysis of the correlation between miR-223 and EPB41L3. (c) Assessment of the binding relation between miR-223 and EPB41L3 using the dual-luciferase reporter gene experiment. (d) Expression level of EPB41L3 in cervical cancer cells after miR-223 expression regulation. (e) Expression of EPB41L3 in tumor and normal tissues based on the CESC-TCGA database.

It was known that EPB41L3 expression level was decreased in several types of carcinomas [25, 26] including cervical cancers. In the current study, we also conducted the analysis about the expression level of EPB41L3 by using the CESC-TCGA database (mentioned above). The results indicated that the expression level of EPB41L3 was low in cervical cancer (Figure 6(e)). Furthermore, we conducted the analysis upon the potential target predictive genes of miR-223 by using TargetScan. As demonstrated in Figure 6(a), EPB41L3 was predicted to be a target gene of miR-223. Additionally, results of the dual-luciferase assay depicted that luciferase activity decreased after transfected with miR-223 (Figures 6(b) and 6(c)). Moreover, EPB41L3 expression level in HeLa cells and Siha cells was tested after the regulation of miR-223, and EPB41L3 expression was inhibited after upregulation of miR-223 (Figure 6(d), Figure S3). In conclusion, the results indicated that miR-223 targets and binds to EPB41L3 in cervical cancer.

### 2.6. Regulation of Downregulated EPB41L3 or miR-223 Mimic Reverses the Effects of Upregulated HDAC10 in Biological Function in Cervical Cancer Cells

**Figure 7:**
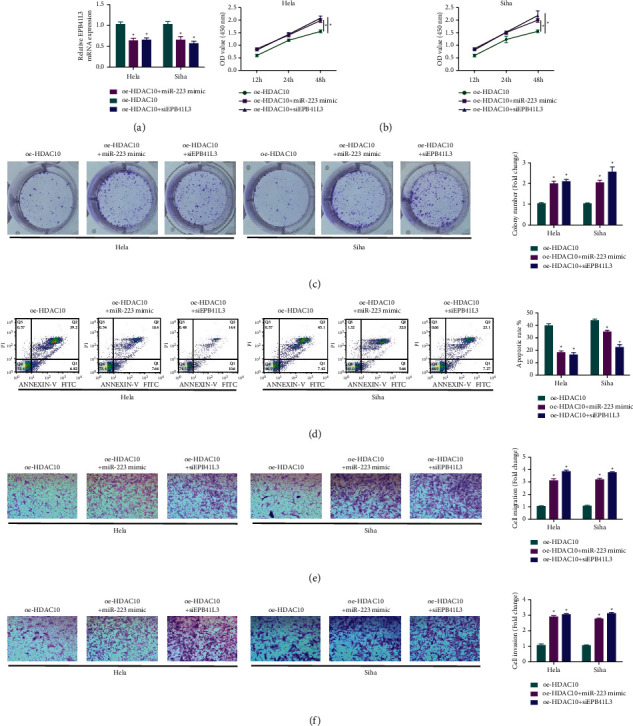
Downregulating EPB41L3 or upregulating miR-223 reverses the biological effects of HDAC10 upregulation in cervical cancer cells. (a) Expression level of EPB41L3 in cervical cancer cells. (b) Detecting cervical cancer cell viability after various treatment. (c) Analysis of the colony-forming abilities of cervical cancer cells. (d) Apoptosis of cervical cancer cells after different treatments. (e). Measurement of cervical cancer cell migration ability. (f) Measurement of cervical cancer cell invasion ability.

For investigating the carcinogenic effects of HDAC10 mediated by miR-223/EPB41L3 axis, we conducted the analysis upon the expression level of EPB41L3 in cells after the treatment of HDAC10 overexpression or after the treatment of miR-223 mimic transfection. The results indicated that the upregulation of HDAC10 would enhance the upregulation effects on EPB41L3. Meanwhile, the upregulated miR-223 could reverse the above effect, which means that the expression level of EPB41L3 was decreased (Figure 7(a)). Furthermore, miR-223 mimic or downregulation of EPB41L3 functionally reversed the effects of upregulation of HDAC10 upon the cervical cancer cells, including the cell viability (Figure 7(b)), colony-forming ability (Figure 7(c)), apoptosis (Figure 7(d)), cell migration (Figure 7(e)), and cell invasion (Figure 7(f)).

## 3. Discussion

With the tumorigenesis of cervical cancer being widely elucidated, together with the increasing vaccination of HPV vaccine [27], the morbidity of cervical cancer decreases year by year [28]. However, cervical carcinoma is still the second most frequently diagnosed gynecological carcinoma in the world. [29]. As the therapies which target the immune checkpoint molecule against cervical cancer have been emerging in the past decade [30, 31], the effectiveness is now reaching a plateau [32, 33]. Thus, understanding and figuring out the influence of HDACs on the cervical cancer progression would contribute to develop the potential preferable therapeutic treatment. Numerous evidences in previous studies reported that HDAC10 was expressed at different levels in diverse kinds of cancers [34]. HDAC10 was reported to negatively regulate the progression of cervical cancer by miR-1908 [9]. Also, poor HDAC10 expression was linked to oncogenesis in cervical cancer via targeting microRNAs and the metastasis via targeting the expression of matrix metalloproteinase [8].

In this present study, we explored the vital role of HDAC10 in suppressing the development of tumorigenesis in cervical cancer. The HDAC10 expression level is downregulated in cervical carcinoma tissues compared to normal cervical tissues. HDAC10 inhibited the migration ability and invasion property in vitro. Also, the results demonstrated that HDAC10 inhibited the ability of tumorigenesis in cervical cancer in vivo via downregulating miR-223 and subsequently targeting EPB41L3.

Firstly, we uncovered that the HDAC10 expression level decreased with worse precancer lesion and was low when compared with normal cervical tissues. In addition, our data reveal that cervical cancer cell with overexpressed HDAC10 exhibited cell viability and colony-forming ability, suggesting that HDAC is connected with cancer progression. These results are in accordance with the conclusions from recent studies that HDAC acts as a regulator in tumor via changing the nucleosome conformation of tumor cells. It is well acknowledged that HADC participates in cellular modulation in various cells by regulating miRNAs, for example, HDAC2 regulates miR-503-5p in esophageal squamous cell carcinoma [35], HDAC2 promotes the endothelial dysfunction induced by diabetes [36, 37], and HDACs regulate the cellular differentiation in neurodegenerative diseases [38].

It was widely acknowledged that microRNAs are small noncoding RNAs with 20–22 nucleotides. MicroRNAs play vital roles in interacting with mRNAs subsequently leading to mRNA degradation or translational repression via silencing targeted genes. It was widely recorded that miRNAs act as biologic regulators or tumorigenesis regulators, in the way of silencing mRNA translation via interacting with mRNAs. MicroRNA-223 was reported as a vital tumor regulator in many cancers [11,39], and it could promote lung cancer cell invasion [40] and influence the ability of migration and invasion via moderating the expression level of artemin (ARTN) [12, 41]. MicroRNA-223 is also involved in regulating the hepatoma cell proliferation through affecting IGF-1R [42] or regulated the metastasis of cervical carcinoma by modulating epithelial-mesenchymal transition [43].

In this study, the results indicate that the expression of miR-223 was high in cervical cancer tissues. Meanwhile, the results indicated that the expression of miR-223 was positively correlated with precancer lesions, which means that miR-223 was highly expressed in worse cervical precancer lesions. Furthermore, HDAC10 binds to miR-223, suggesting a novel regulation pathway that depends on acetylation. Moreover, our previous study showed that the severity degree of precancer lesions was related to EPB41L3, a kind of possible cancer suppressor protein. The results in this study showed that miR-223 targets EPB41L3, and EPB41L3 reversed the effects of miR-223.

## 4. Conclusion

In conclusion, our study provides evidence that HDAC10 downregulates the expression miR-223 in cervical cancer and subsequently targets EPB41L3. The pathway plays a role in tumor regulation in cervical cancer and provides novel insights upon the management of cervical cancer or precancer lesions.

## 5. Materials and Methods

### 5.1. Ethics Approval

This study was approved by the Institutional Review Board of the Obstetrics and Gynecology Hospital of Fudan University. All the study process was implemented based on the Declaration of Helsinki. Also, the study obtained the oral informed consents and the written informed consents. Also, we acquired a survey from the enrolled participants for the demographic data collection. All animal experiments were housed and maintained in a standard environment, and the protocol was reviewed and approved by the Animal Care and Research Committee of Fudan University.

### 5.2. Cell Culture

Human cervical cancer cell lines and human normal vaginal epithelial cell were acquired from the laboratory of the Obstetrics and Gynecology Hospital of Fudan University. Cells were cultured using Dulbecco's modified Eagle's medium (DMEM) with 10% fetal bovine serum, as well as 100 U/mL penicillin and 100 *μ*g/mL amycin.

### 5.3. Cell Transfection Assay

The cell transfection assay was conducted by using the lentivirus targeting human EPB41L3 (si-EPB41L3, GenePharma, Shanghai, China). Also, the negative control was selected as the corresponding nontargeting negative control (si-NC). Briefly, we transfected miR-223 NC (Ribobio, Guangzhou, China), miR-223 mimic (Ribobio, Guangzhou, China), and pcDNA-HDAC10 (Ribobio, Guangzhou, China) into cervical cancer cells.

### 5.4. Cell Viability Assay

We measured the cell viability via using a Cell Counting Kit-8 (CCK-8; Dojindo Laboratories, Kumamoto, Japan). An amount of 5 × 10^3^ cervical cancer cells were seeded in 96-well plates overnight. Then, we added 10 *μ*l CCK-8 reagent into each well and cultured the system at 37°C for another 1 hour, which was followed by measurement using a spectrophotometer at OD450.

### 5.5. Colony Formation Assay

Cervical cancer cells were cultured and seeded in the 6-well plates (Corning, NY, USA) at the concentration of 500 cells/well. Then, cells were cultured at 37°C for 2 weeks. Cells were fixed with paraformaldehyde (4% v/v) for 30 minutes and stained with 0.1% crystal violet solution for 1 hour.

### 5.6. Flow Cytometric Analysis of Apoptosis

We used Phycoerythrin (PE) Annexin V Apoptosis Detection Kit I (BD Biosciences) to analyze the apoptosis. After cervical cancer cells were cultured for 24 h, cells were washed three times with PBS and resuspended in 1X binding buffer. Then, we added 5 *μ*l PE Annexin V and 5 *μ*l 7-amino-actinomycin (7-AAD) into the binding buffer followed by the incubation at room temperature. Thereafter, apoptotic cells were detected via flow cytometry (FV500, Beckman Coulter, Brea, USA). We used the FlowJo 7.6 software (FlowJo, LLC, Ashland, OR) to analyze the data. Each experiment was conducted in triplicate.

### 5.7. Migration Assay

Cervical cancer cells (HeLa and Siha) were starved for 12 hours in a serum-free medium. Totally, 10^5^ cells were seeded into the upper transwell chamber with 200 *μ*l of serum-free medium (Corning, New York), and totally, 600 *μ*l medium with 10% FBS was added to the lower chamber. After cultured at 37°C for 12 hours, cells that migrated to the underside were fixed via 4% paraformaldehyde for 40 minutes, followed by the staining via 0.1% crystal violet for 10 minutes. Finally, an upright metallurgical microscope was used to photograph five random fields in the membrane underside, and cells that had migrated were counted.

### 5.8. Invasion Assay

Cervical cancer cells (HeLa and Siha) were starved for 12 hours in a serum-free medium. The insert chamber was pretreated with 50ul Matrigel. Totally, 1 × 10^5^ cells were seeded into the upper chamber, and 500 *μ*l medium (supplemented with 10% FBS) was added into the lower chamber. After cultured at 37°C for 24 hours, invaded cells through the filter pores to the underside of the insert were fixed with 4% paraformaldehyde for 30 minutes, followed by staination with 0.1% crystal violet for 10 minutes. Finally, an upright metallurgical microscope was used to photograph five random fields in the membrane underside, and cells that had successfully invaded were counted. All experiments were repeated in triplicate.

### 5.9. Chromatin Immunoprecipitation (ChIP) Assay

Cells were crosslinked using 1% formaldehyde and then lysed and subsequently underwent sonication to shear DNA. The ChIP assay was conducted by using a ChIP Assay Kit (Millipore, USA). Also, the antibodies were prepared as follows: anti-HDAC10 (1:100, sc-81599) from Santa Cruz (Santa Cruz, USA), and the negative control was normal IgG (ab172730) from Abcam.

### 5.10. Data Analysis

In this study, we conducted the correlation analysis not only for the gene expression evaluation but also for the survival prognosis evaluation in cervical carcinoma, which is based on the TCGA database (https://tcga-data.nci.nih.gov). Furthermore, we conducted analysis (including the expression analysis and the survival analysis) upon the HDAC10 and EPB41L3 based on the GEPIA2 website (http://gepia2.cancer-pku.cn/).

### 5.11. Participant Enrollment and Specimen Collections

In the current study, 10 women with cervical cancer and 10 patients who underwent hysterectomy with noncancer disease were enrolled in the study between February 2019 and February 2020. Also, cervical scraping samples were collected in a colposcopy center from 20 cases of NILM, 20 cases of LSIL, 20 cases of HSIL, and 20 cases of cervical cancer. All of the cases had histopathological diagnosis and the oral and written consents were obtained.

### 5.12. Statistical Analysis

The SPSS 25.0 software was selected for the statistical analysis. Data were presented as mean ± standard deviation. T tests were used for comparisons of means between two groups; meanwhile, one-way ANOVA tests were used for comparisons of more than two groups. Statistical analysis was conducted by using SPSS 25.0 (IBM, NY) and the Excel software. The statistical significance was defined as *p* value <0.05.

## Figures and Tables

**Table 1:** Demographic and clinical characteristics of the participants.

	Histology				*p* value
	Normal (*n* = 20)	LSIL (*n* = 20)	HSIL (*n* = 20)	Cervical cancer (*n* = 20)	
Characteristics					
Age					
<30 y	11	10	8	8	0.382
≥30 y	9	10	12	12	
Marital status					
Steady partner*∗*	15	16	16	15	0.071
No steady partner*∗∗*	5	4	4	5	
Smoker					
No	3	3	4	2	0.064
Yes	17	17	16	18	
Number of sexual partners					
0-1	19	17	18	1	0.032
≥2	1	3	2	19	

*∗*represents married women or women with a stable partner; *∗∗*represents single women, widows, or divorcees.

**Table 2:** Primer sequence.

Primer		
EPB41L3	Sense	5′-GAGCTGCCAAGCGTTTATGGA-3′
	Antisense	5′-CCTGCCACTATAACGAAACTTGGAA-3′

miR-223	Sense	5′-CAGAAAGCCCAATTCCATCT -3′
	Antisense	5′-GGGCAAATGGATACCATACC-3′

HDAC10	Sense	5′-CGATGTGTAGCCCATAGAGGT-3′
	Antisense	5′-CCACAGAATTCTCCCATTGC-3′

U6	Sense	5′-CTCGCTTCGGCAGCACA-3′
	Antisense	5′-AACGCTTCACGAATTTGCGT-3′

GAPDH	Sense	5′-CAAGGTCATCCATGACAACTTTG-3′
	Antisense	5′-GTCCACCACCCTGTTGCTGTAG-3′

## Data Availability

The full datasets are not publicly available due to the need to protect participant confidentiality; however, the data that support the findings of this study are available on request from the corresponding author. Inquiries should be communicated to the corresponding author who will consider all sufficiently specified and reasonable requests.

## Supplementary Materials

Supplementary Materials Fig S1: photographs of the original western blot membranes for HDAC10. (A) The samples from left to right: normal tissues, cervical cancer tissues, and the protein bands from top to bottom: HDAC19 (75 kDa). (B) The samples from left to right: normal tissues, cervical cancer tissues, and the protein bands from top to bottom: actin (42 kDa). Cervical cancer tissues expressed lower protein levels of HDAC10 compared to normal tissues. Figure S1 shows representative membranes for the data shown in Figure 1(d). Fig S2: photographs of the original western blot membranes for AcH3K9. (A) The samples from left to right: oe-NC, oe-HDAC10, and the protein bands from top to bottom: AcH3K9 (17 kDa) and H3 (15 kDa). Cervical cancer Siha cells after overexpressing HDAC10 expressed lower protein levels of AcH3K9 than the control. Figure S2 shows representative membranes for the data shown in Figure 5(c). Fig S3: photographs of the original western blot membranes for EPB41L3. (A) The samples from left to right: miR-223-NC, miR-223-mimic, and the protein bands from top to bottom: EPB41L3 (120 kDa). (B) The samples from left to right: miR-223-NC, miR-223-mimic, and the protein bands from top to bottom: actin (42 kDa). Cervical cancer cells after upregulating miR-223 expressed lower protein levels of EPB41L3 compared to the control treatment. Figure S3 shows representative membranes for the data shown in Figure 6(d).
