# Supplementary figures and images for: HDAC10 Inhibits Cervical Cancer Progression through Downregulating the HDAC10-microRNA-223-EPB41L3 Axis
Source: J Oncol. 2022 Jan 19;2022:8092751. doi: 10.1155/2022/8092751 (PMC8783137; doi:10.1155/2022/8092751)

**A**

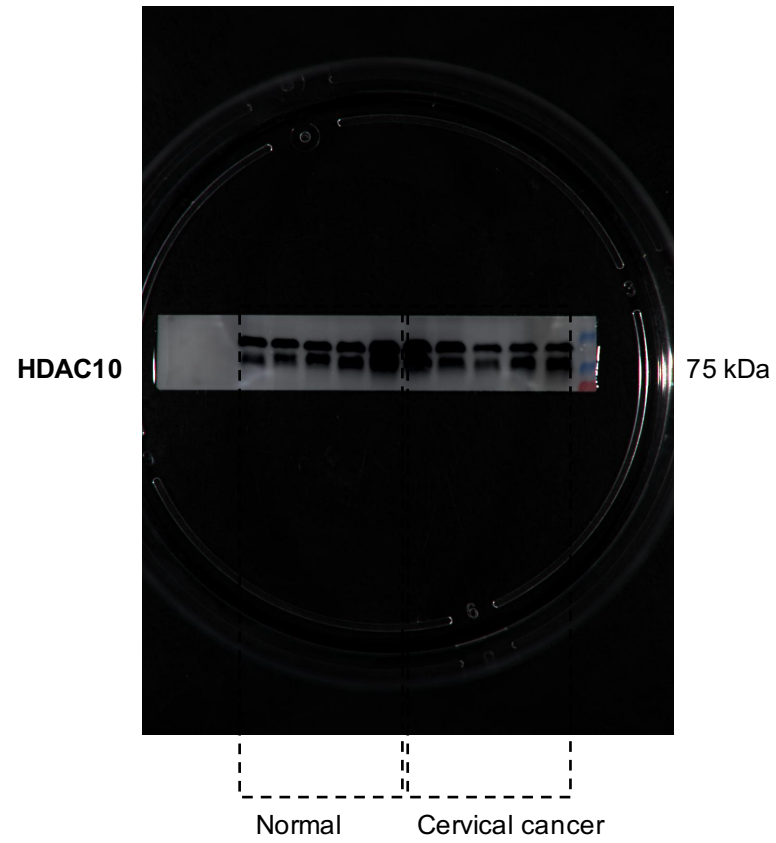

**B**

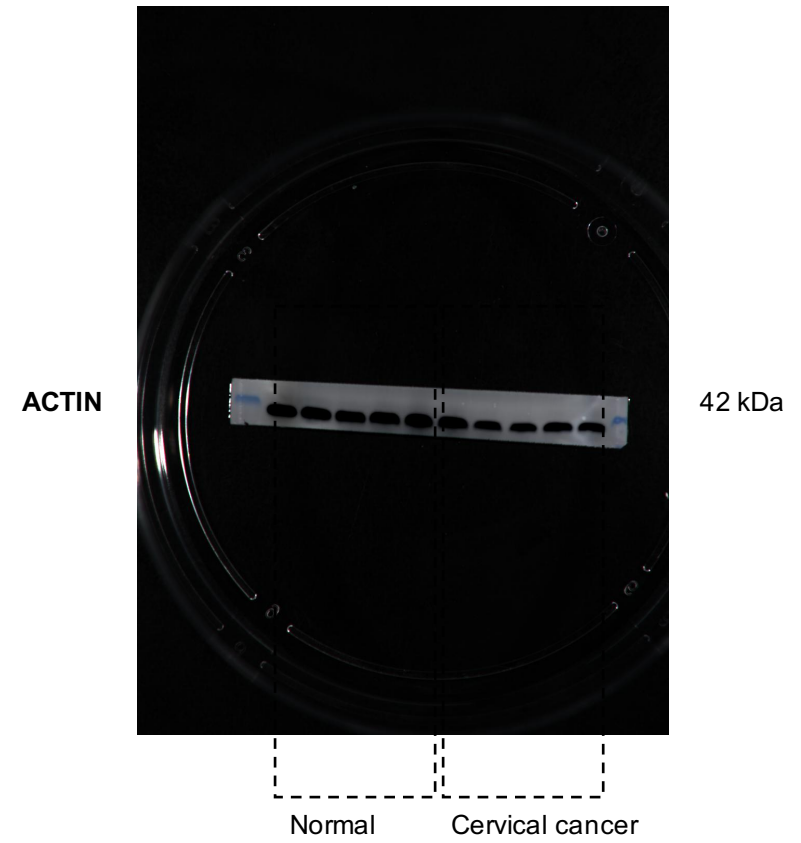

Supplement: Supplementary Materials — Fig S1: photographs of the original western blot membranes for HDAC10. (A) The samples from left to right: normal tissues, cervical cancer tissues, and the protein bands from top to bottom: HDAC19 (75 kDa). (B) The samples from left to right: normal tissues, cervical cancer tissues, and the protein bands from top to bottom: actin (42 kDa). Cervical cancer tissues expressed lower protein levels of HDAC10 compared to normal tissues. Figure S1 shows representative membranes for the data shown in Figure 1(d). Fig S2: photographs of the original western blot membranes for AcH3K9. (A) The samples from left to right: oe-NC, oe-HDAC10, and the protein bands from top to bottom: AcH3K9 (17 kDa) and H3 (15 kDa). Cervical cancer Siha cells after overexpressing HDAC10 expressed lower protein levels of AcH3K9 than the control. Figure S2 shows representative membranes for the data shown in Figure 5(c). Fig S3: photographs of the original western blot membranes for EPB41L3. (A) The samples from left to right: miR-223-NC, miR-223-mimic, and the protein bands from top to bottom: EPB41L3 (120 kDa). (B) The samples from left to right: miR-223-NC, miR-223-mimic, and the protein bands from top to bottom: actin (42 kDa). Cervical cancer cells after upregulating miR-223 expressed lower protein levels of EPB41L3 compared to the control treatment. Figure S3 shows representative membranes for the data shown in Figure 6(d). [file 8092751.f1.zip › 8092751.f1/Fig S1.pdf]

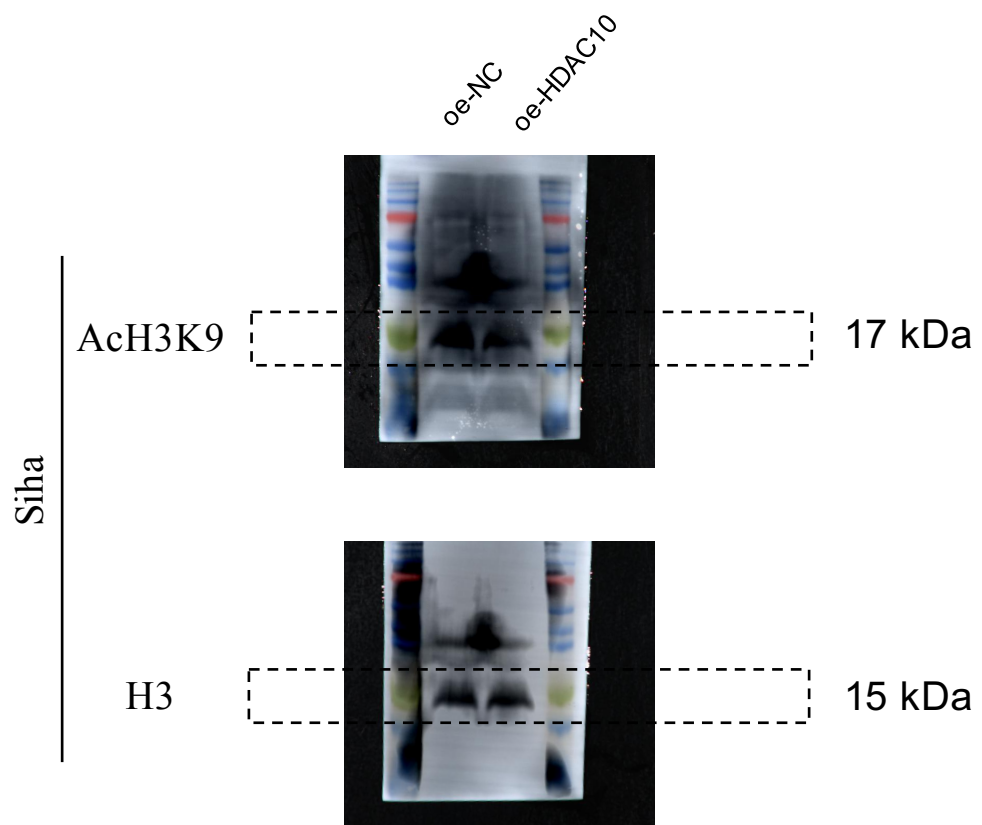

Supplement: Supplementary Materials — Fig S1: photographs of the original western blot membranes for HDAC10. (A) The samples from left to right: normal tissues, cervical cancer tissues, and the protein bands from top to bottom: HDAC19 (75 kDa). (B) The samples from left to right: normal tissues, cervical cancer tissues, and the protein bands from top to bottom: actin (42 kDa). Cervical cancer tissues expressed lower protein levels of HDAC10 compared to normal tissues. Figure S1 shows representative membranes for the data shown in Figure 1(d). Fig S2: photographs of the original western blot membranes for AcH3K9. (A) The samples from left to right: oe-NC, oe-HDAC10, and the protein bands from top to bottom: AcH3K9 (17 kDa) and H3 (15 kDa). Cervical cancer Siha cells after overexpressing HDAC10 expressed lower protein levels of AcH3K9 than the control. Figure S2 shows representative membranes for the data shown in Figure 5(c). Fig S3: photographs of the original western blot membranes for EPB41L3. (A) The samples from left to right: miR-223-NC, miR-223-mimic, and the protein bands from top to bottom: EPB41L3 (120 kDa). (B) The samples from left to right: miR-223-NC, miR-223-mimic, and the protein bands from top to bottom: actin (42 kDa). Cervical cancer cells after upregulating miR-223 expressed lower protein levels of EPB41L3 compared to the control treatment. Figure S3 shows representative membranes for the data shown in Figure 6(d). [file 8092751.f1.zip › 8092751.f1/Fig S2.pdf]

**A**

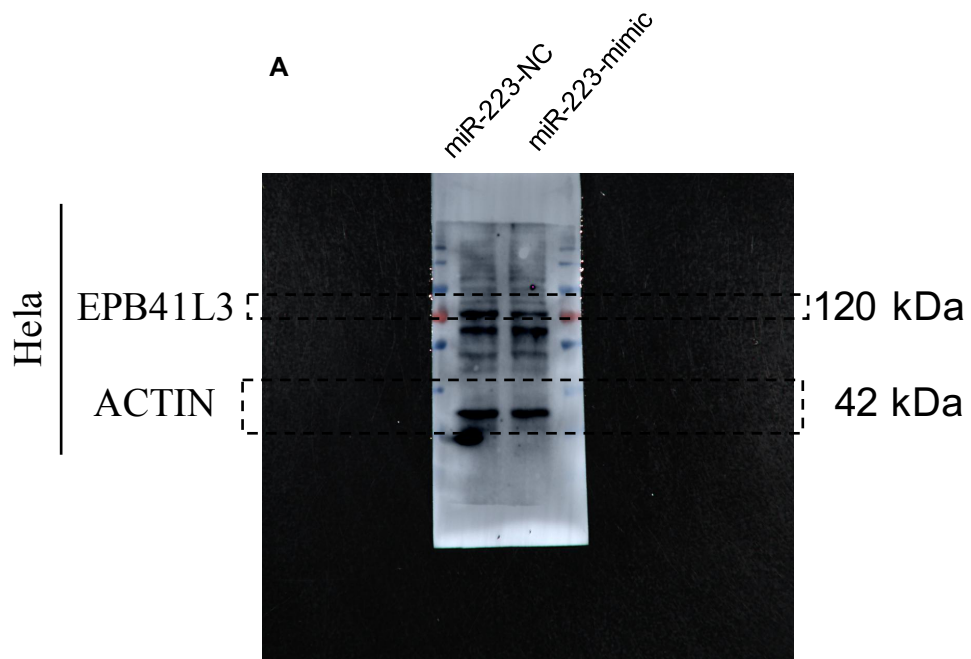

**B**

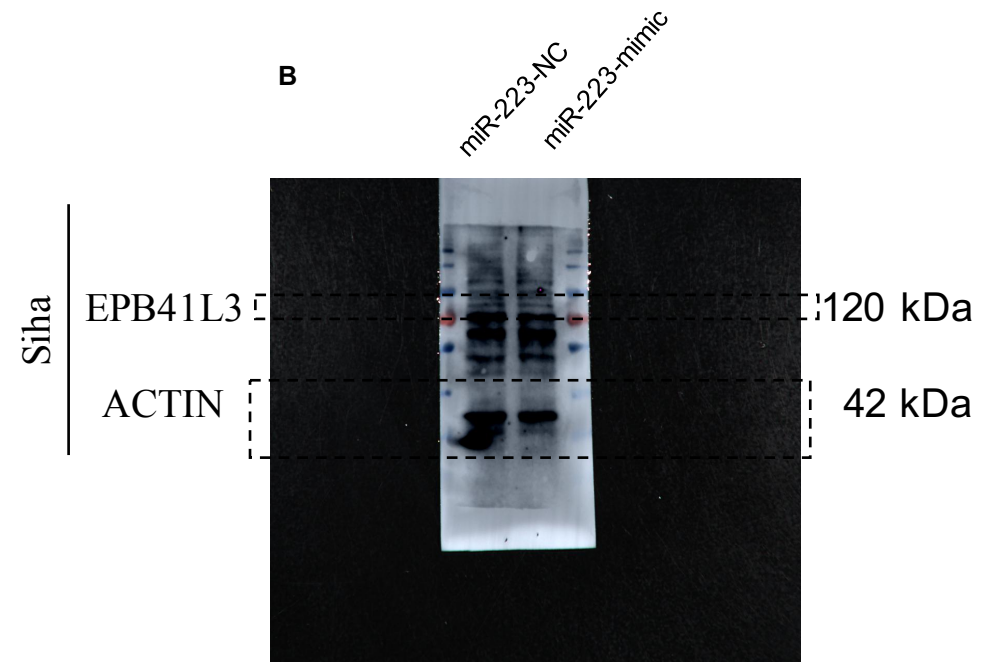

Supplement: Supplementary Materials — Fig S1: photographs of the original western blot membranes for HDAC10. (A) The samples from left to right: normal tissues, cervical cancer tissues, and the protein bands from top to bottom: HDAC19 (75 kDa). (B) The samples from left to right: normal tissues, cervical cancer tissues, and the protein bands from top to bottom: actin (42 kDa). Cervical cancer tissues expressed lower protein levels of HDAC10 compared to normal tissues. Figure S1 shows representative membranes for the data shown in Figure 1(d). Fig S2: photographs of the original western blot membranes for AcH3K9. (A) The samples from left to right: oe-NC, oe-HDAC10, and the protein bands from top to bottom: AcH3K9 (17 kDa) and H3 (15 kDa). Cervical cancer Siha cells after overexpressing HDAC10 expressed lower protein levels of AcH3K9 than the control. Figure S2 shows representative membranes for the data shown in Figure 5(c). Fig S3: photographs of the original western blot membranes for EPB41L3. (A) The samples from left to right: miR-223-NC, miR-223-mimic, and the protein bands from top to bottom: EPB41L3 (120 kDa). (B) The samples from left to right: miR-223-NC, miR-223-mimic, and the protein bands from top to bottom: actin (42 kDa). Cervical cancer cells after upregulating miR-223 expressed lower protein levels of EPB41L3 compared to the control treatment. Figure S3 shows representative membranes for the data shown in Figure 6(d). [file 8092751.f1.zip › 8092751.f1/Fig S3.pdf]
